# PD1 ligand functionality a biomarker of response to anti PD1 treatment in patients with HNSCC

**DOI:** 10.1038/s41698-024-00620-y

**Published:** 2024-06-03

**Authors:** Bar Kaufman, Tarek Taha, Orli Abramov, Yaniv Zohar, Kamel Mhameed, Ofir Cohen, Angel Porgador, Moshe Elkabets, Salem Billan

**Affiliations:** 1 The Shraga Segal Department of Microbiology, Immunology and Genetics, Faculty of Health Sciences, Ben-Gurion University of the Negev, Beer-Sheva, 84105 Israel; 2 The Joseph Fishman Oncology Center, Rambam Health Care Campus, Haifa, Affiliated to the Rappaport Faculty of Medicine, Israel Institute of Technology-Technion, Haifa, 3109601 Israel; 3 Oncology institute, Tzafon Medical Center, Poriya affiliated with Azrieli Faculty of Medicine, Bar Ilan University, Ramat Gan, Israel; 4 Institute of Pathology, Rambam Health Care Campus, Haifa, 3109601 Israel

**Keywords:** Diagnostic markers, Head and neck cancer

## Abstract

Therapies targeting the PD-1/PD-L1 pathway have transformed head and neck squamous cell carcinoma (HNSCC) treatment. However, predicting the response to anti-PD-1 therapy remains a clinical challenge. This study evaluated the functional binding of PD-1 ligands in 29 HNSCC patients and compared it to the standard PD-L1 Combined Positive Score (CPS). The assessment of PD-1 ligands’ functionality advances the current ability to predict the response of HNSCC patients to anti-PD-1 therapy.

## Introduction

Immune-checkpoint inhibitors (ICIs), including those targeting PD-1/PD-L1^1^, have revolutionized immuno-oncology, significantly improving overall survival (OS)^2,3^ and quality of life^4^. Despite anti-PD-1-based improvement in the prognosis of head and neck squamous cell carcinoma (HNSCC)^5^, challenges persist due to the modest response rate^6,7^, emphasizing the need for a predictive biomarker to enhance outcomes in precision immuno-oncology^8^. Various biomarkers linked to the anti-PD-1 response have been explored in HNSCC^9^, including the Combined Positive Score (CPS) of PD-L1 staining^10,11^, measuring the proportion of PD-L1-positive cells in a tumor. However, the response rate among CPS-positive patients is poor^11^, although some CPS-negative patients respond to anti-PD-1 treatment^12^.

Recently, we introduced a novel approach^13^ – designated Immune checkpoint Artificial Reporter overexpressing PD-1 (IcAR-PD-1) – for assessing PD-1 ligand (PD-L1^14^ and PD-L2^15^) functionality to predict anti-PD-1 response. Our current evaluation, conducted on HNSCC patient samples, investigates the association between PD-1 ligand functionality and CPS-based PD-L1 abundance. The study cohort included 29 patients (Table 1) receiving anti-PD-1 therapy at Rambam Medical Center from 2017 to 2022. The cohort was chosen from a total of 57 patients, such that the intentional diversity ensured equal representation of responders and non-responders, with different treatment lines and CPS statuses. Fifteen patients were non-responders (stable or progressive disease), and 14, responders (partial or complete response). There were no significant differences between the two groups in demographics, diagnostic features, or treatment details, except for mortality (43% in responders vs. 80% in non-responders, *p* = 0.039).

**Table. 1:** Cohort description

Characteristics	Overall *N* = 29^a^	Non-responders *N* = 15^a^	Responders *N* = 14^a^	*p*-value^b^
**Age**	65 (9)	65 (9)	66 (10)	*0.9*
**Sex**				*0.8*
Male	11 / 29 (38%)	6 / 15 (40%)	5 / 14 (36%)	
Female	18 / 29 (62%)	9 / 15 (60%)	9 / 14 (64%)	
**Ethnicity**				*0.3*
Jewish	24 / 29 (83%)	11 / 15 (73%)	13 / 14 (93%)	
Arab	5 / 29 (17%)	4 / 15 (27%)	1 / 14 (7.1%)	
**Exitus**	18 / 29 (62%)	12 / 15 (80%)	6 / 14 (43%)	*0.039*
**Smoking**	16 / 29 (55%)	7 / 15 (47%)	9 / 14 (64%)	*0.3*
**Metastasis**				*0.6*
Metastatic	4 / 29 (14%)	3 / 15 (20%)	1 / 14 (7.1%)	
Local	25 / 29 (86%)	12 / 15 (80%)	13 / 14 (63%)	
**CPS**				*0.2*
<1%	11 / 28 (39%)	7 / 15 (47%)	4 / 13 (31%)	
1–20%	10 / 28 (36%)	3 / 15 (20%)	7 / 13 (54%)	
>20%	7 / 28 (25%)	5 / 15 (33%)	2 / 13 (15%)	
**Leukocytes**	6.9 (4.2)	8.4 (4.5)	5.3 (3.5)	*0.2*
**Neutrophils**	5.2 (3.8)	6.2 (4.4)	4.0 (2.9)	*0.4*
**Lymphocytes**	1.08 (0.60)	1.22 (0.51)	0.93 (0.67)	*0.3*
**Albumin**	2.29 (1.90)	2.87 (1.61)	1.66 (2.06)	*0.2*
**Treatment protocol**				*0.2*
Durvalumab	1 / 29 (3.4%)	1 / 15 (6.7%)	0 / 14 (0%)	
Nivolumab	5 / 29 (17%)	3 / 15 (20%)	2 / 14 (14%)	
Pembrolizumab	20 / 29 (69%)	11 / 15 (73%)	9 / 14 (64%)	
Pembrolizumab+carboplatin+ paclitaxel	3 / 29 (10%)	0 / 15 (0%)	3 / 14 (21%)	
**Line of treatment**				*0.7*
1	10 / 29 (34%)	4 / 15 (27%)	6 / 14 (43%)	
2	17 / 29 (59%)	10 / 15 (67%)	7 / 14 (50%)	
3	2 / 29 (6.9%)	1 / 15 (6.7%)	1 / 14 (7.1%)	
**Number of cycles**	13 (13)	6 (8)	21 (13)	*<0.001*
**Progression-free survival (PFS)**	17 (21)	7 (15)	27 (21)	*<0.001*
**Overall survival (OS)**	24 (21)	17 (18)	32 (21)	*0.036*

^a^Mean (SD); *n/N* (%).

^b^Wilcoxon rank sum test; Pearson’s Chi-squared test; Fisher’s exact test; Wilcoxon rank sum exact test.

Selected cohort of 29 out of 57 HNSCC patients treated at Rambam Medical Center from 2017 to 2022. The cohort was intentionally selected to ensure equal representation of responders and non-responders, based on RECIST criteria, and hence encompassing diverse lines of anti-PD-1 treatment and CPS statuses.

Most patients (25/29) received anti-PD-1 monotherapy (20 receiving pembrolizumab and 5, nivolumab); three were treated with an anti-PD-1 combination of carboplatin and paclitaxel; and one received anti-PD-L1 treatment (durvalumab). There was no significant difference in the distribution of treatments between the two groups (chi-square, *p* = 0.2). Of the cohort, 10, 17, and 2 received the above treatments as first-, second-, and third-line treatments, respectively, with no significant difference in the distribution of the line of treatment between the two groups (chi-square, *p* = 0.7). However, the average number of treatment cycles differed significantly between the groups (Wilcoxon rank, *p* < 0.001), with an average of 6 cycles for non-responders and a significantly higher average of 21 cycles for responders.

**Fig. 1:**
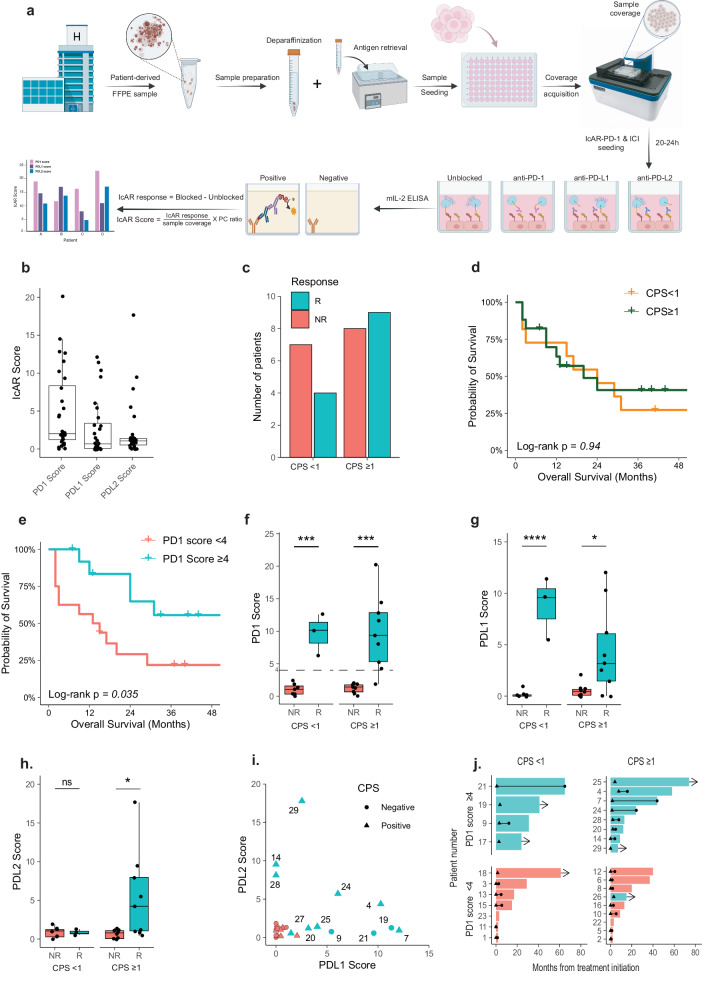
Functional PD-1 ligand binding demonstrates a stronger association with anti-PD-1 therapy response compared to CPS. **a** Schematic representation of the scientific process for analyzing patient samples (Created with BioRender.com). **b** Boxplots illustrating different PD-1 ligand scores calculated for individual patients in our cohort (*n* = 29). **c** Distribution of the cohort into responders (R) and non-responders (NR) based on CPS status (*n* = 28). **d** Survival curves indicating non-significant differences in overall survival time between responders and non-responders (log-rank *p* = 0.94, *n* = 28) based on CPS. **e** Survival curves showing significant differences in overall survival time between responders and non-responders (log-rank p = 0.035, *n* = 29) based on PD-1 score. **f–h** Boxplots displaying PD-1, PD-L1, and PD-L2 scores for responders (R) and non-responders (NR), segregated by CPS status. **i** Dot-plot depicting the relationship between PD-L1 and PD-L2 scores. CPS status is indicated by the shape of the dots: triangles for CPS positive and circles for CPS negative. **j**. Swimmer plots showing the time from treatment initiation layered by CPS status and PD-1 score; teal = responder, red = non-responder, triangle = beginning of the response, circle = end of the response, line = duration of response, arrow = ongoing response.

To evaluate PD-1 ligand functionality, FFPE samples were placed in a 96-well plate, and tissue coverage was measured with a JuliStage histo-recorder (Fig. 1a). After co-culture with IcAR-PD-1 cells, PD-L1 and PD-L2 binding was assessed, and ligand-receptor interactions were disrupted using antibodies. Functionality scores (PD-1, PD-L1, and PD-L2) were assigned to each patient (Fig. 1b). In the CPS-negative group (CPS ≤ 1), only 36% responded, while the CPS-positive group (CPS > 1) exhibited a response rate of 53% (Fig. 1c, Table 1). PD-L1 CPS did not significantly stratify patients based on OS (Fig. 1d), but PD-1 and PD-L1 scores, with a cutoff of 4, showed significant OS separation (Fig. 1e and Supplementary Fig. 1a). Higher PD-1 scores consistently correlated with positive responses across subgroups (Fig. 1f). The PD-L1 score exhibited a similar pattern, but PD-L2 score patterns were specific to positive CPS cases (Fig. 1g, h). Analyzing individual patients revealed varied responses (Fig. 1i). Responders often had high PD-L1 scores, while some, particularly positive CPS cases (e.g., Patients 14 and 28), showed functional PD-L2 and low PD-L1 functionality. Patients 24 and 4 displayed both functional PD-L1 and PD-L2. Those with exclusive functional PD-L1 showed no discernible trend in CPS status. Stratified analysis by both PD-1 score and CPS (Fig. 1j) emphasized that patients with a PD-1 score above 4 demonstrated a higher overall response rate, with 5 of 12 achieving an OS exceeding 40 months. Additionally, patients with a PD-1 score above 4 exhibited more ongoing responses, with 4 of 12 patients responding, compared to 2 of 16 patients in the PD-1 score <4 group. Cross-validation and cohort sampling tests demonstrated robust predictive performance of the Functionality scores for clinical response, with a minimum test AUC of 0.89 across cohort sizes (Supplementary Fig. 1b, c).

**Fig. 2:**
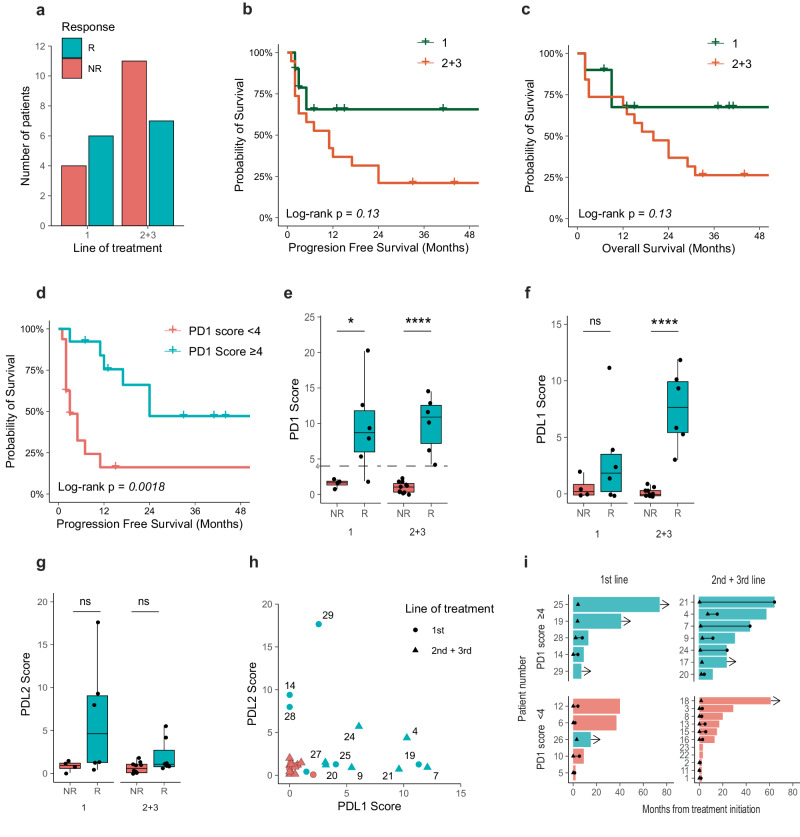
Functional PD-1 ligand binding associates with response to anti-PD-1 therapy, independent of treatment line. **a**. Cohort distribution into responders (R) and non-responders (NR) based on treatment lines. **b**. Survival curves indicating non-significant differences in progression-free survival time between responders and non-responders (log-rank *p* = 0.13, *n* = 29) based on the line of treatment. **c**. Survival curves indicating non-significant differences in overall survival time between responders and non-responders (log-rank *p* = 0.13, *n* = 29) based on line of treatment. **d**. Survival curves showing significant differences in progression-free survival time between responders and non-responders (log-rank *p* = 0.0018, *n* = 29) based on PD1 score. **e-g**. Boxplots displaying PD-1, PD-L1, and PD-L2 scores for responders (R) and non-responders (NR), segregated by line of treatment. **h** Dot-plot depicting the relationship between PD-L1 and PD-L2 scores. The line of treatment is indicated by the shape of the dots: triangles for 1st line and circles for 2nd and 3rd lines. **i** Swimmer plots showing the time from treatment initiation layered by treatment lines and PD1 score; teal = responder, red = non-responder, triangle = beginning of response, circle= end of response, line = duration of response, arrow = ongoing response.

To investigate the correlation between treatment lines, treatment response, and PD-1 ligand functionality in our cohort, we narrowed our focus to 19 out of the patients who received anti-PD-1 as second- or third-line treatment vs. the 10 patients who received it as first-line treatment. Initial examination indicated a response rate of 60% (6/10) for first-line anti-PD-1 therapy and 43% (8/11) for second- or third-line treatment (Fig. 2a). Despite a seemingly greater benefit in first-line treatment, no significant differences were observed in patient progression-free survival (PFS) (Fig. 2b) or OS (Fig. 2c) based on the line of treatment. Stratification by a PD-1 and PD-L1 scores of 4 revealed distinct PFS patterns (Fig. 2d and Supplementary Fig. 1d), with PFS being significantly longer for patients with scores above 4. Further analysis showed a clear differentiation in clinical response based on PD-1 scores across different treatment lines (Fig. 2e). Most patients (92%) with PD-1 scores above 4 responded to treatment, including 5 of 6 first-line recipients and all 6 s- or third-line patients.

The ability to differentiate responders from non-responders differed for PD-L1 and PD-L2 scores. The PD-L1 score distinguished between second- and third-line recipients (Fig. 2f), while the PD-L2 score showed no differentiation across treatment lines (Fig. 2g). Detailed analysis of individual patients’ PD-L1 and PD-L2 scores (Fig. 2h) highlighted specific patterns: Patients with primarily functional PD-L2 were often first-line recipients, whereas those displaying mainly functional PD-L1 were predominantly second- or third-line recipients. Patients exhibiting both functional PD-L1 and PD-L2 were typically second- or third-line recipients (Fig. 2i). An in-depth examination of response patterns (Fig. 2j), considering both PD-1 score and treatment line, revealed significant trends. Patients with a PD-1 score above 4 had a higher overall response rate, with 5 of 12 reaching an OS exceeding 40 months. In terms of PFS, first-line recipients with a PD-1 score above 4 displayed ongoing responses more frequently than those with a score below 4. Patients with a PD-1 score above 4 also showed a significantly longer average response duration (28 months) compared with those with a score below 4 (8 months) (*p* = 0.006).

This is the first clinical study in HNSCC to use an artificial reporter-based assay as a means of measuring the functionality of PD-1 ligands and of associating them with patient response to anti-PD1 treatment^13^. Comparing IcAR-PD1 to current predictive methods revealed a significant correlation between PD-1 ligands functionality and treatment response, potentially surpassing CPS for stratifying patients. A noteworthy 92% of patients (12/13) with a PD-1 score above 4 responded positively, surpassing 52% (9/17) with a positive CPS. Interestingly, 36% of patients (4/11) with a negative CPS also responded. Both methods crucially assess tumor and surrounding non-tumor cells^16^, acknowledged contributors to the PD1-PDL1/2 axis. IcAR-PD1 is distinguished by its ability to evaluate ligand-receptor interaction and quantify PD-L1 and PD-L2 functionality, highlighting a key difference to CPS staining: Notably, ligand abundance (CPS PD-L1 staining) differs from functionality (IcAR), as demonstrated in our work^13^. While other predictive methods, such as gene expression analysis, have shown potential for response prediction^17^, their complexity, cost, and limitations for use with FFPE samples restrict their widespread use, in contrast to IcAR-PD1, which is potentially more compatible with routinely collected FFPE tissue.

We further examined the robustness of our findings by stratifying patients according to the line of treatment. Despite the lack of statistical significance, there was a trend suggesting first-line recipients are more likely to respond. Ligand-specific IcAR scores revealed possible explanations for different treatment lines: patients expressing high functional PD-L2, crucial for HNSCC outcomes^18,19^, were mainly among first-line recipients, while of those in the PD-L1 positive PD-L2 negative group, a notable 71%, were in the second- and third-line groups. Our findings align with existing knowledge^11,20,21^ that the initial line of chemotherapy can modify the inhibitory landscape of the tumor and tumor microenvironment by enhancing PD-L1 levels, and such alterations can potentially enhance the PD-L1-mediated responsiveness of tumors to anti-PD-1 therapy.

The emergence of new ICI treatments underscores the need for predictive biomarkers across a broader immunotherapy spectrum. Following the IcAR-PD1 reporter, additional reporters for various immune checkpoints (ICs) could predict the success of mono or combined ICI therapy. We envision a future where each patient’s IC-based landscape would assist physicians in selecting the optimal ICI treatment plan. While promising, our study does have the limitations of small sample size and potential cohort mismatch, emphasizing the need for collaborative, nationwide studies for broader insights into anti-PD-1 therapy response in HNSCC.

In conclusion, this pilot study underscores the potential for assessing the functionality of PD-1 ligands to enhance the precision of predicting patient response to anti-PD-1 therapy. Our findings demonstrate that IcAR outperforms the current method, CPS, used for stratifying patients based on their response to treatment. As we continue to advance the field of precision oncology, function-based methodologies will play a pivotal role in enhancing patient outcomes and transforming the landscape of immuno-oncology.

## Methods

### FFPE Co-Culture and Evaluation of IcAR Activation

Five 5-µm FFPE sections were combined and deparaffinized using HistoChoice® (#H2779, Sigma-Aldrich). After gradual rehydration, antigen retrieval was performed in Tris-EDTA buffer, and the samples were then suspended in 1X PBS and seeded in a 96-well plate. Surface area coverage was analyzed using Juli™ Stat software. For co-culture, 100,000 IcAR-PD-1 cells were seeded on FFPE samples for 24 h. IcAR-PD-1 receptor, PD-L1, and PD-L2 were blocked with 10 µg/mL of pembrolizumab, durvalumab, and anti-human CD273 (clone MIH18), respectively. Supernatants were collected after 24 h, and murine IL-2 levels were assessed using a commercial ELISA kit. The ELISA plates were pre-coated, blocked, and incubated with supernatants, followed by detection using streptavidin-horseradish peroxidase conjugate and TMB.

### Calculation of IcAR Score

IL-2 measurements in each well were normalized by dividing them by the log2-transformed sample coverage, yielding the IL-2 signal per log2(mm^2^). The IcAR score for each sample well, calculated separately for each antibody using Eq. (1), included the PC ratio for normalization across experiments. Sample coverage was obtained using Juli™ Stage and analyzed with the Juli™ Stat software.

1 $${IcAR\; score}=\frac{\triangle {IcAR\; response}}{{{Log}}_{2}({Sample\; coverage})}* {PC\; ratio}$$

where $$\Delta\,{IcAR\; response}={Unblocked\;response}\,\left({no\; AB}\right)-{Blocked\;response}\,\left({PD}1\,{or\; PDL}1\right)$$

### Combined Positive Score (CPS)

CPS for programmed death-ligand 1 (PD-L1) staining quantifies PD-L1 expression in tumor and immune cells within the tumor microenvironment. It involves summing the percentages of PD-L1-positive cells, dividing by the total viable tumor cells, and multiplying by 100. A CPS value ≥ 1% signifies positivity, offering a comprehensive assessment of PD-L1 expression.

### Clinical evaluation of patients

Response to treatment was assessed using the RECIST 1.1 criteria, which involved monitoring changes in tumor size through baseline and regular imaging scans during treatment. Responders were identified as patients achieving a complete response (CR) or partial response (PR) according to RECIST criteria. Non-responders included patients with stable disease (SD) or progressive disease (PD) based on RECIST criteria. Overall survival was defined as the duration from diagnosis to death or last follow-up. For deceased patients, survival time was capped at the date of death, while for living patients, survival time was capped at the last follow-up. Progression-free survival (PFS) was defined as the time elapsed between treatment initiation and tumor progression or death from any cause.

### Calculation of the predictive power of IcAR score

Three IcAR features—PDL1, PDL2, and PDL2 IcAR score—were utilized to develop a predictive model using Extreme Gradient Boosting (XGBoost). The model was trained with a binary label distinguishing Responders from Non-Responders. To prevent overfitting, we limited XGBoost optimization to a single round. Performance evaluation was conducted using the XGBoost function customized for cross-validation. The cohort was split into training and test sets using 3-fold cross-validation (2/3 training, 1/3 testing) via the xgb.cv function. This analysis was repeated across 100 random simulated cohorts drawn from our original cohort of 29 patients. To examine the relationship between cohort size and model performance, we iterated this process with sampled cohorts ranging from 15 to 29 individuals.xs

### Statistical analysis

All statistical analyses were performed using the R programming language (version 4.2.3). The data were first cleaned and preprocessed, after which descriptive statistics were calculated. Inferential statistics were then applied to test the hypotheses of the study. Differences were considered to be statistically significant at a two‐sided *P* < 0.05. The specific packages and functions used for each analysis are available upon request.

### Reporting summary

Further information on research design is available in the Nature Research Reporting Summary linked to this article.

### Supplementary information

Supplementary Fig. 1

Reporting Summary

## Data Availability

All data supporting the findings of this study are available within the paper and its Supplementary Information.

## Code availability

The Machine Learning analysis to assess the accuracy of our IcAR features and power analysis was performed with Extreme Gradient Boosting (XGBoost) as implemented in the R package xgboost (Version 1.7.7.1). The classification was based on the binary label of Responders vs. Non-Responders with a single round of XGBoost optimization. We assessed performance with 3-fold cross-validation using xgb.cv function. Boxplot visualization was done with ggplot2 (Version 3.4.4). The raw data and R code used in this study is available upon request.
