# Supplementary material for: PD1 ligand functionality a biomarker of response to anti PD1 treatment in patients with HNSCC
Source: NPJ Precis Oncol. 2024 Jun 3;8:126. doi: 10.1038/s41698-024-00620-y (PMC11148182; doi:10.1038/s41698-024-00620-y)
Supplement: Supplementary file 1 — Supplementary Fig. 1 [file 41698_2024_620_MOESM1_ESM.pdf]

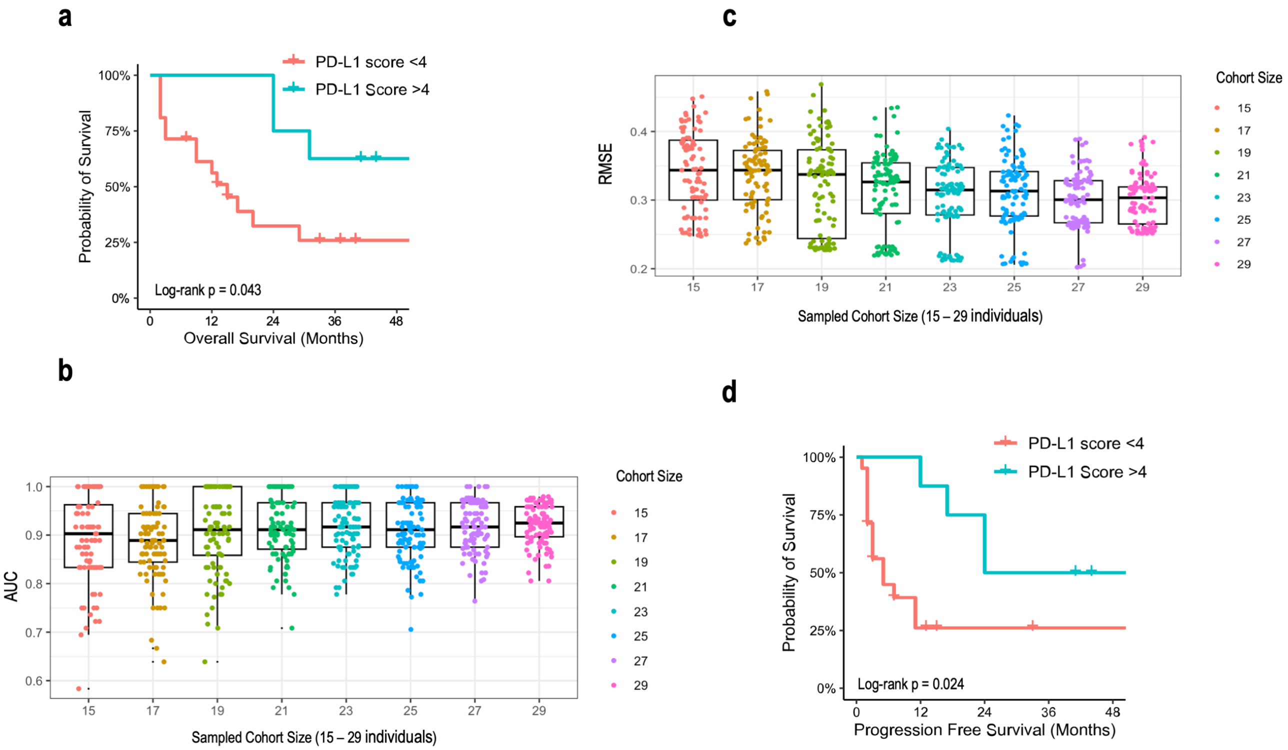

Supplementary Fig. 1. Performance of the model and survival analysis a. Survival curves showing significant differences in overall survival time between responders and non-responders (log-rank p = 0.043, n = 29) based on PD-L1 score. b. Area under the ROC Curve (AUC) shown by mean test-AUC (solid line) and its standard deviation (shaded area) across 100 randomly simulated cohorts of varying sizes (x-axis) sampled from the original cohort of 29 patients. The y-axis represents the AUC value. Higher AUC indicates better model performance. (Mean test AUC = 0.9068, range = 0.92 – 0.89, SD = 0.04 – 0.091.) c. Root mean squared error (RMSE) shown by mean test-RMSE (solid line) across 100 randomly simulated cohorts of varying sizes (x-axis) sampled from the original cohort of 29 patients. The y-axis represents the RMSE value. Lower RMSE indicates better model performance. (Mean test RMSE = 0.316, range = 0.3 – 0.343.) d. Survival curves showing significant differences in progression free survival time between responders and non-responders (log-rank p = 0.024, n = 29) based on PD-L1 score.
