# Supplementary material for: PD1 ligand functionality a biomarker of response to anti PD1 treatment in patients with HNSCC
Source: NPJ Precis Oncol. 2024 Jun 3;8:126. doi: 10.1038/s41698-024-00620-y (PMC11148182; doi:10.1038/s41698-024-00620-y)
Supplement: Supplementary file 2 — Reporting Summary [file 41698_2024_620_MOESM2_ESM.pdf]

nature portfolio

Corresponding author(s): Moshe Elkabets and Salem Bilan

Last updated by author(s): Mar 26, 2024

Reporting Summary

Nature Portfolio wishes to improve the reproducibility of the work that we publish. This form provides structure for consistency and transparency in reporting. For further information on Nature Portfolio policies, see our Editorial Policies and the Editorial Policy Checklist.

Statistics

For all statistical analyses, confirm that the following items are present in the figure legend, table legend, main text, or Methods section.

n/a	Confirmed	
☐	☒	The exact sample size (*n*) for each experimental group/condition, given as a discrete number and unit of measurement
☐	☒	A statement on whether measurements were taken from distinct samples or whether the same sample was measured repeatedly
☐	☒	The statistical test(s) used AND whether they are one- or two-sided<br>*Only common tests should be described solely by name; describe more complex techniques in the Methods section.*
☐	☒	A description of all covariates tested
☐	☒	A description of any assumptions or corrections, such as tests of normality and adjustment for multiple comparisons
☒	☐	A full description of the statistical parameters including central tendency (e.g. means) or other basic estimates (e.g. regression coefficient) AND variation (e.g. standard deviation) or associated estimates of uncertainty (e.g. confidence intervals)
☒	☐	For null hypothesis testing, the test statistic (e.g. *F*, *t*, *r*) with confidence intervals, effect sizes, degrees of freedom and *P* value noted<br>*Give P values as exact values whenever suitable.*
☒	☐	For Bayesian analysis, information on the choice of priors and Markov chain Monte Carlo settings
☒	☐	For hierarchical and complex designs, identification of the appropriate level for tests and full reporting of outcomes
☒	☐	Estimates of effect sizes (e.g. Cohen's *d*, Pearson's *r*), indicating how they were calculated

Our web collection on statistics for biologists contains articles on many of the points above.

Software and code

Policy information about availability of computer code

Data collection	No software were used
Data analysis	R Studio: Libraries: survival,survminer, dplyr, ggplot2, swimplot, reshape2, gtsummary, ggrepel

For manuscripts utilizing custom algorithms or software that are central to the research but not yet described in published literature, software must be made available to editors and reviewers. We strongly encourage code deposition in a community repository (e.g. GitHub). See the Nature Portfolio guidelines for submitting code & software for further information.

Data

Policy information about availability of data

All manuscripts must include a data availability statement. This statement should provide the following information, where applicable:

- Accession codes, unique identifiers, or web links for publicly available datasets
- A description of any restrictions on data availability
- For clinical datasets or third party data, please ensure that the statement adheres to our policy

All data supporting the findings of this study are available within the paper and its Supplementary Information.

Research involving human participants, their data, or biological material

Policy information about studies with human participants or human data. See also policy information about sex, gender (identity/presentation), and sexual orientation and race, ethnicity and racism.

Reporting on sex and gender	The authors report that there is no significant difference in the distribution of sex between responders and non-responders, with similar proportions observed in both groups (Table 1)
Reporting on race, ethnicity, or other socially relevant groupings	The authors report that there is no significant difference in the distribution of ethnicity between responders and non-responders, with similar proportions observed in both groups (Table 1).
Population characteristics	The authors report that there is no significant difference in the distribution of age and smoking status between responders and non-responders, with similar proportions observed in both groups (Table 1).
Recruitment	The study cohort consisted of 29 patients (Table 1, Supplemental Fig. 1a-b) who received anti-PD-1 therapy at Rambam Medical Center between 2017 and 2022, selected from a total of 57 patients. The deliberate diversity in patient selection ensured equal representation of responders and non-responders, encompassing various treatment lines and CPS statuses. Additionally, there were no significant demographic differences observed among the patients.
Ethics oversight	Rambam Health Care Campus

Note that full information on the approval of the study protocol must also be provided in the manuscript.

Field-specific reporting

Please select the one below that is the best fit for your research. If you are not sure, read the appropriate sections before making your selection.

☒ Life sciences ☐ Behavioural & social sciences ☐ Ecological, evolutionary & environmental sciences

For a reference copy of the document with all sections, see nature.com/documents/nr-reporting-summary-flat.pdf

Life sciences study design

All studies must disclose on these points even when the disclosure is negative.

Sample size	29
Data exclusions	No
Replication	All attempts of replication were successful
Randomization	The study cohort included 29 patients (Table 1) receiving anti-PD-1 therapy at Rambam Medical Center from 2017 to 2022. The cohort was chosen from a total of 57 patients, such that the intentional diversity ensured equal representation of responders and non-responders, with different treatment lines and CPS statuses.
Blinding	The investigators were blinded to group allocation during all phases of the study, including data collection, experiment process, and data analysis.

Reporting for specific materials, systems and methods

We require information from authors about some types of materials, experimental systems and methods used in many studies. Here, indicate whether each material, system or method listed is relevant to your study. If you are not sure if a list item applies to your research, read the appropriate section before selecting a response.

Materials & experimental systems

n/a	Involved in the study	
☐	☒	Antibodies
☐	☒	Eukaryotic cell lines
☒	☐	Palaeontology and archaeology
☒	☐	Animals and other organisms
☐	☒	Clinical data
☒	☐	Dual use research of concern
☒	☐	Plants

Methods

n/a	Involved in the study	
☒	☐	ChIP-seq
☒	☐	Flow cytometry
☒	☐	MRI-based neuroimaging

Antibodies

Antibodies used	pembrolizumab, durvalumab, and anti-human CD273 (clone MIH18)
Validation	The antibodies were obtained in their original packaging. Further analysis involved validating Pembrolizumab by staining IcAR-PD1 cells and blocking PD1-PDL1/2 interaction. Durvalumab was validated by blocking IcAR-PD1 interaction with FC-rhPDL1-coated wells. Additionally, anti-human CD273 Durvalumab was validated by blocking IcAR-PD1 interaction with his-rhPDL2-coated wells.

Eukaryotic cell lines

Policy information about cell lines and Sex and Gender in Research

Cell line source(s)	BW-5147
Authentication	Non of the cell lines in use were authenticated
Mycoplasma contamination	All cell lines used for this study were tested and found negative for Mycoplasma contamination
Commonly misidentified lines (See ICLAC register)	*Name any commonly misidentified cell lines used in the study and provide a rationale for their use.*

Clinical data

Policy information about clinical studies

All manuscripts should comply with the ICMJE guidelines for publication of clinical research and a completed CONSORT checklist must be included with all submissions.

Clinical trial registration	0695-21-RMB
Study protocol	Study protocol could be found at Rambam Health Care Campus protocol refference: 0695-21-RMB
Data collection	This retrospective study was conducted on FFPE samples obtained from patients diagnosed with Head and Neck Squamous Cell Carcinoma (HNSCC).
Outcomes	Primary outcome was to asses PD-1 ligand functionallity in samples derived from HNSCC patients

Plants

Seed stocks	*Report on the source of all seed stocks or other plant material used. If applicable, state the seed stock centre and catalogue number. If plant specimens were collected from the field, describe the collection location, date and sampling procedures.*
Novel plant genotypes	*Describe the methods by which all novel plant genotypes were produced. This includes those generated by transgenic approaches, gene editing, chemical/radiation-based mutagenesis and hybridization. For transgenic lines, describe the transformation method, the number of independent lines analyzed and the generation upon which experiments were performed. For gene-edited lines, describe the editor used, the endogenous sequence targeted for editing, the targeting guide RNA sequence (if applicable) and how the editor was applied.*
Authentication	*Describe any authentication procedures for each seed stock used or novel genotype generated. Describe any experiments used to assess the effect of a mutation and, where applicable, how potential secondary effects (e.g. second site T-DNA insertions, mosiacism, off-target gene editing) were examined.*
